# Diagnosis of a Neonate With Long QT Syndrome and Severe Complications Delayed due to an Unrecognized Familial History

**DOI:** 10.1155/crpe/5621484

**Published:** 2025-11-30

**Authors:** Kaho Kawabata, Ayako Chida-Nagai, Kenta Takeda, Ryota Honjo, Daisuke Sasaki, Hirokuni Yamazawa, Yosuke Kaneshi

**Affiliations:** ^1^Department of Pediatrics, Hokkaido University, Sapporo, Japan; ^2^Department of Neonatology, Sapporo City General Hospital, Sapporo, Japan

**Keywords:** arrythmia, intracranial hemorrhage, long QT syndrome, multidisciplinary communication, neonate

## Abstract

Long QT syndrome (LQTS) is a hereditary arrhythmic disorder associated with sudden cardiac death. We report a neonatal case of congenital LQTS that went undiagnosed in utero, leading to severe postnatal complications. A male preterm infant was delivered by emergency cesarean section due to fetal hydrops. Shortly after birth, he developed respiratory failure and metabolic acidosis, followed by ventricular tachycardia and torsades de pointes requiring prolonged resuscitation. During cardiopulmonary resuscitation, the father collapsed, and it was subsequently established that he had been receiving treatment for LQTS. The paternal grandmother also had the same diagnosis. Genetic testing performed for the infant identified a pathogenic *KCNH2* variant (c.1714G > A, p.Gly572Ser), confirming Type 2 LQTS. Despite antiarrhythmic therapy and ventricular pacing, the patient developed severe intraventricular hemorrhage and hydrocephalus. This case serves to emphasize the importance of obtaining a detailed family history and the need for effective communication among obstetricians, neonatologists, and cardiologists. In this instance, a lack of awareness regarding familial LQTS contributed to a delay in diagnosis and intervention. Our findings highlight the essential roles played by early detection and prenatal risk assessment through family screening and genetic testing for preventing life-threatening arrhythmia and improving outcomes in congenital LQTS.

## 1. Introduction

Long QT syndrome (LQTS) is an inherited condition associated with sudden death due to life-threatening arrhythmias [1, 2]. In this paper, we present a case of congenital LQTS, wherein the presence of the disease went undetected during fetal development, subsequently leading to severe complications and prolonged cardiopulmonary resuscitation from birth.

## 2. Case Presentation

The mother, a 28-year-old primigravida, experienced an uneventful pregnancy until 28 weeks and 6 days of gestation, at which time she noticed a reduction in fetal movement. Ultrasound monitoring performed at an obstetric clinic revealed pleural and ascitic fluid, prompting an emergency transfer to our hospital. Transient fetal bradycardia and reduced movement were observed, necessitating emergency cesarean section. Prior to birth, the developing fetus had not been examined by a fetal cardiology specialist.

At birth, the infant weighed 1391 g and had generalized edema, with Apgar scores of 5 and 8 at 1 and 5 min, respectively. The infant was unable to sustain independent breathing, thus necessitating intubation and surfactant administration at 3 min after birth. Blood test results revealed metabolic acidosis. An echocardiogram was performed immediately after birth, which revealed no congenital structural abnormalities. The infant's condition deteriorated with bradycardia, and catecholamine infusion was initiated in the neonatal intensive care unit (NICU). At 6 h postbirth, the patient experienced ventricular tachycardia (VT) that progressed to torsades de pointes, necessitating prolonged cardiopulmonary resuscitation and frequent defibrillation.

**Figure 1:**
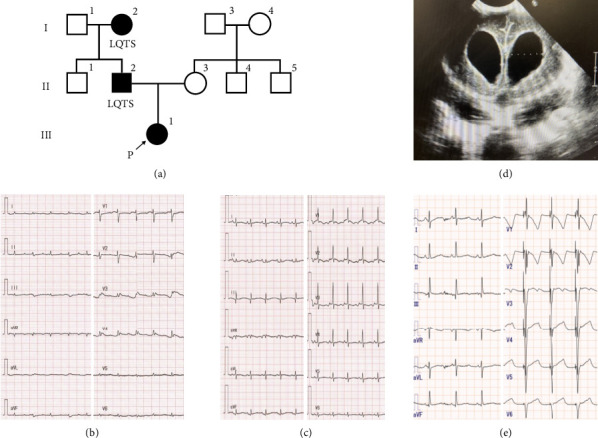
Family pedigree and clinical findings. (a) Family pedigree: The patient's family history was not disclosed to the neonatal intensive care unit (NICU) medical staff until the father experienced a syncopal episode in the NICU. (b) 12-lead electrocardiogram taken on Day 0 of life, during treatment with propranolol. The patient exhibited a prolonged Fridericia-corrected QT interval of 0.533 s. (c) Electrocardiogram on Day 17 of life. The patient was receiving continuous intravenous landiolol and mexiletine; the corrected QT interval by Fridericia's formula had shortened to 0.371 s. (d) Cranial ultrasonography on Day 41 shows progressive hemorrhagic hydrocephalus. (e) Following implantable cardioverter–defibrillator implantation and administration of oral mexiletine and propranolol, an electrocardiogram obtained at 18 months of age showed marked QT prolongation (Fridericia-corrected QT interval of 0.542 s).

Having been informed of his child's condition, the father visited the NICU, during which the infant developed sustained VT and was subjected to multiple episodes of electrical defibrillation. The father soon reported feeling unwell and shortly after collapsed. Immediate evaluation was initiated, including blood pressure and electrocardiographic assessments. No abnormal findings were detected, and he gradually regained consciousness. However, further investigation revealed that the father has LQTS and was on antiarrhythmic therapy. Moreover, it was established that the infant's paternal grandmother also had LQTS (Figure 1(a)).

Based on this family history, the infant was suspected of having LQTS, and, consequently, continuous infusion of landiolol was initiated, in response to which a 12-lead electrocardiogram revealed a prolonged corrected QT time (Figure 1(b)).

However, VT persisted, necessitating the addition of intravenous mexiletine. Temporary pacing leads were implanted on Day 1. Although atrial and ventricular epicardial leads were placed, the atrial lead subsequently became dislodged, which was suspected to be ascribed to chest compressions performed during the procedure. Consequently, only the ventricular lead was used, and pacing was maintained in VVI mode. However, although this procedure effectively prevented fatal arrhythmia, head ultrasonography on Day 3 revealed a Grade 3 intraventricular hemorrhage and progressive hemorrhagic hydrocephalus. On Day 17, QT interval shortening was observed on an electrocardiogram after administration of mexiletine (Figure 1(c)). On Day 43, an Ommaya reservoir was placed for cerebrospinal fluid drainage (Figure 1(d)), and at 2 months of age, antiarrhythmic therapy was replaced with oral medications, with propranolol (3 mg/kg/day) and mexiletine (5 mg/kg/day) administered for the subsequent 3 months.

At 4 months of age, ventriculoperitoneal (VP) shunt surgery was deferred, as ventricular fibrillation had been triggered by transfer stress. The plan was to perform cardioverter–defibrillator implantation followed by VP shunt surgery after the child had gained sufficient weight. Following implantable cardioverter–defibrillator implantation, an electrocardiogram obtained at 18 months of age showed marked QT prolongation (Figure 1(e)); however, no arrhythmic events occurred during follow-up.

Genetic testing identified a heterozygous pathogenic variant of *KCNH2* [NM_000238.4], c.1714G > A, p.Gly572Ser, thereby confirming Type 2 LQTS.

## 3. Discussion

Congenital LQTS can cause fetal death and sudden infant death syndrome, and cases of in utero arrhythmias carry a high risk of heart failure, hydrops fetalis, and fetal demise, which often necessitates early delivery [3–5].

The Japanese Circulation Society guidelines on hereditary cardiac diseases recommend genetic testing for suspected LQTS cases based on clinical symptoms and family history to confirm the diagnosis [6]. This is likewise recommended in the United States and Europe [7, 8].

Obtaining a detailed family history is critically important in idiopathic cardiac conditions [9]. However, in the present case, although the patient's father and paternal grandmother had LQTS, the family was unaware of the risk of inheriting this disorder. Moreover, although the father's history was disclosed to the initial obstetrician, it was not thereafter communicated to the pediatric team, thereby contributing to a delay in diagnosis and treatment. The absence of genetic testing in the father was a key factor underlying the initial failure to recognize the disease in the patient. Given that Type 2 LQTS is generally more severe in females than in males [10], the paternal disease history may be overlooked.

Emotional stress and abrupt auditory stimuli are characteristic triggers in Type 2 LQTS [11]. In the present case, witnessing his son's resuscitation may have induced severe psychological stress in the father, who had Type 2 LQTS, thereby triggering a life-threatening arrhythmic event.

Using transplacental beta-blocker therapy, Shima et al. [12] have reported the successful management of fetal arrhythmia and uncomplicated full-term delivery in patients with Type 2 LQTS. In the present case, early suspicion and monitoring may have enabled timely intervention for the detection of arrhythmia.

In conclusion, early diagnosis, prompt treatment, and favorable outcomes of congenital LQTS require comprehensive communication regarding family histories, along with sufficient patient education. Importantly, collaborative efforts among obstetricians, pediatricians, and cardiologists are essential for ensuring the necessary dissemination of information and optimal care.

## Data Availability

The data that support the findings of this study are available on request from the corresponding author. The data are not publicly available due to privacy or ethical restrictions.

## References

[1] Roden D. M., Spooner P. M. (1999). Inherited Long QT Syndromes: a Paradigm for Understanding Arrhythmogenesis. *Journal of Cardiovascular Electrophysiology*.

[2] Horigome H., Nagashima M., Sumitomo N. (2010). Clinical Characteristics and Genetic Background of Congenital long-QT Syndrome Diagnosed in Fetal, Neonatal, and Infantile Life: a Nationwide Questionnaire Survey in Japan. *Circulation, Arrhythmia and Electrophysiology*.

[3] Crotti L., Tester D. J., White W. M. (2013). Long QT syndrome-associated Mutations in Intrauterine Fetal Death. *JAMA*.

[4] Samples S., Cherny S., Madan N. (2025). The Prenatal Diagnosis and Perinatal Management of Congenital Long QT Syndrome: a Comprehensive Literature Review and Recent Updates. *Journal of Cardiovascular Development and Disease*.

[5] Chivers S., Ovadia C., Regan W. (2023). Systematic Review of Long QT Syndrome Identified During Fetal Life. *Heart Rhythm*.

[6] Imai Y., Kusano K., Aiba T. (2024). Japanese College of Cardiology; Japanese Society of Pediatric Cardiology; Cardiac Surgery Joint Working Group JCS/JCC/JSPCCS 2024 Guideline on Genetic Testing and Counseling in Cardiovascular Disease. *Circulation Journal*.

[7] Al-Khatib S. M., Stevenson W. G., Ackerman M. J. (2018). 2017 AHA/ACC/HRS Guideline for Management of Patients with Ventricular Arrhythmias and the Prevention of Sudden Cardiac Death: Executive Summary. *Journal of the American College of Cardiology*.

[8] Priori S. G., Wilde A. A., Horie M. (2013). HRS/EHRA/APHRS Expert Consensus Statement on the Diagnosis and Management of Patients with Inherited Primary Arrhythmia Syndromes: Document Endorsed by HRS, EHRA, and APHRS in May 2013 and by ACCF, AHA, PACES, and AEPC in June 2013. *Heart Rhythm*.

[9] Waddell-Smith K. E., Donoghue T., Oates S. (2016). Inpatient Detection of cardiac-inherited Disease: the Impact of Improving Family History Taking. *Open Heart*.

[10] Migdalovich D., Moss A. J. (2011). Mutation and Gender-specific Risk in Type 2 Long QT Syndrome: Implications for Risk Stratification for life-threatening Cardiac Events in Patients with Long QT Syndrome. *Heart Rhythm*.

[11] Schwartz P. J., Priori S. G., Spazzolini C. (2001). Genotype-Phenotype Correlation in the long-QT Syndrome: Gene-Specific Triggers for life-threatening Arrhythmias. *Circulation*.

[12] Shima Y., Horigome H., Nozaki Y. (2020). Successful Trans-maternal Nadolol Pharmacotherapy in a Fetus Presenting with Long QT Syndrome Type 2 Complicated by Torsade de Pointes. *Journal of Cardiology Cases*.

